# Supplementary material for: linc-mipep and linc-wrb encode micropeptides that regulate chromatin accessibility in vertebrate-specific neural cells
Source: eLife. 2023 May 16;12:e82249. doi: 10.7554/eLife.82249 (PMC10188112; doi:10.7554/eLife.82249)
Supplement: Supplementary file 8. [file elife-82249-supp8.pdf]

Supplementary Figure 1. ATAC peak intensity plots for statistically different peaks between wild type and *linc-mipep1* cells. Per cluster: top, heat-map of chromatin accessibility by peak (blue scale increasing in accessibility); bottom, P value of statistically different chromatin accessibility peaks between wild type and mutant cells, using Wilcoxon rank sum (red) and the Kolmogorov-Smirnov (blue) methods using one-tailed tests for each condition. Raw p-value thresholds of 0.001 and 0.01 for the Wilcoxon and the KS tests, respectively, were deemed to be significant peaks for further analysis.

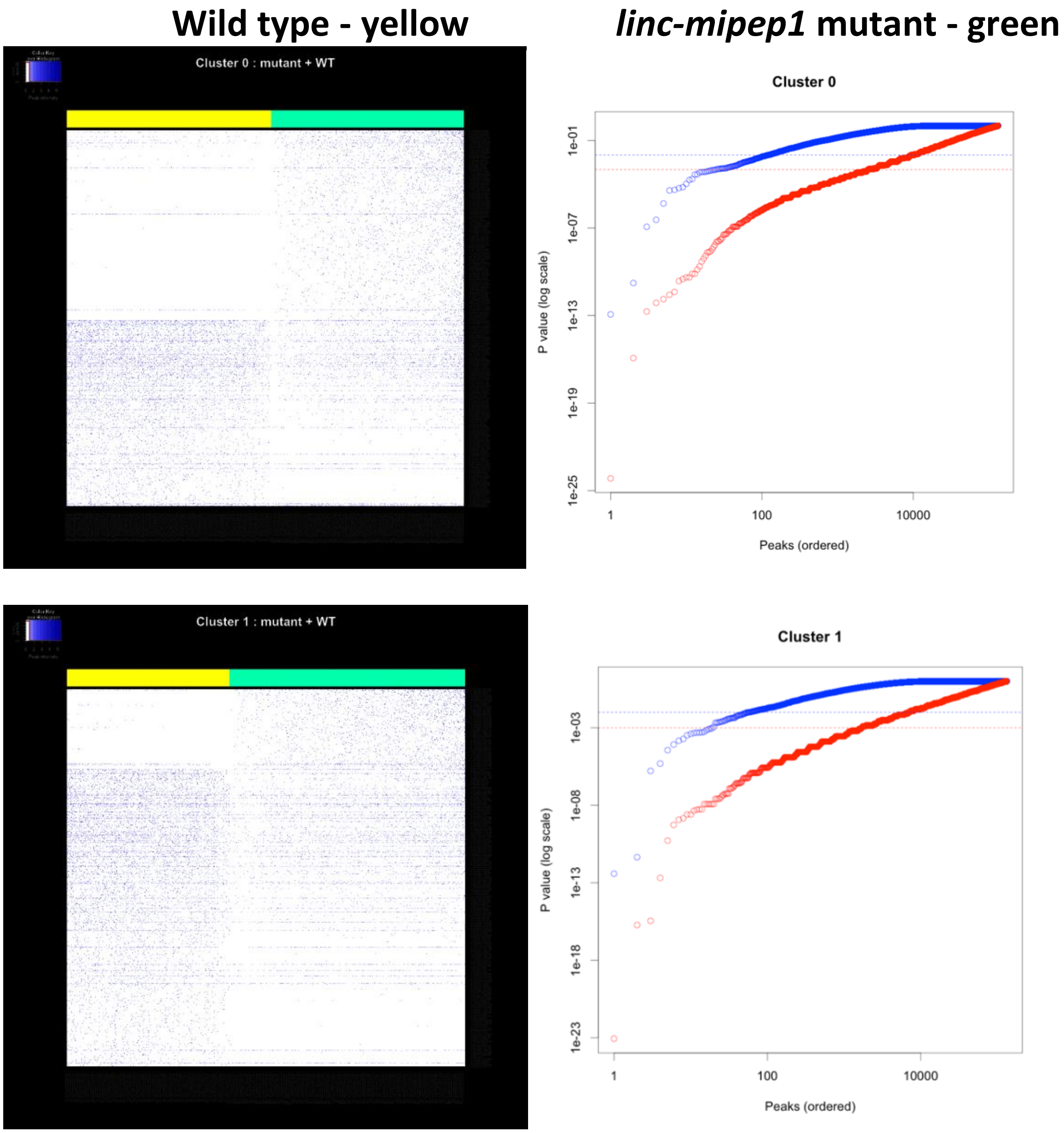

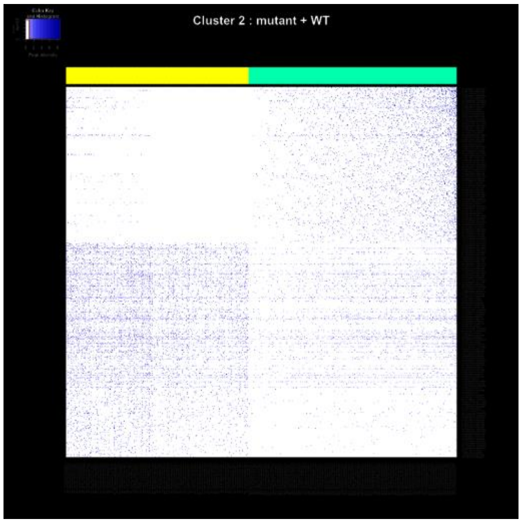
Cluster 2 : mutant + WT

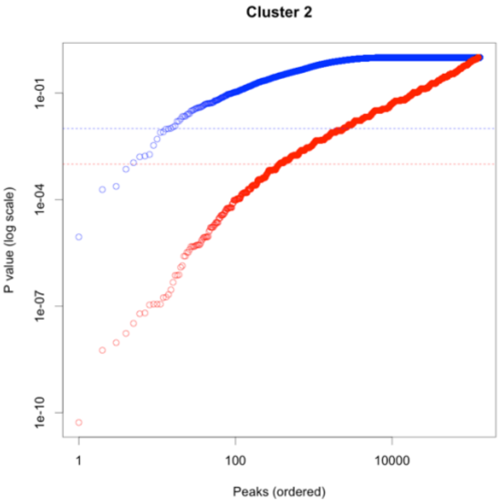
Cluster 2
1e-01
1e-04
1e-07
1e-10
P value (log scale)
1
100
10000
Peaks (ordered)

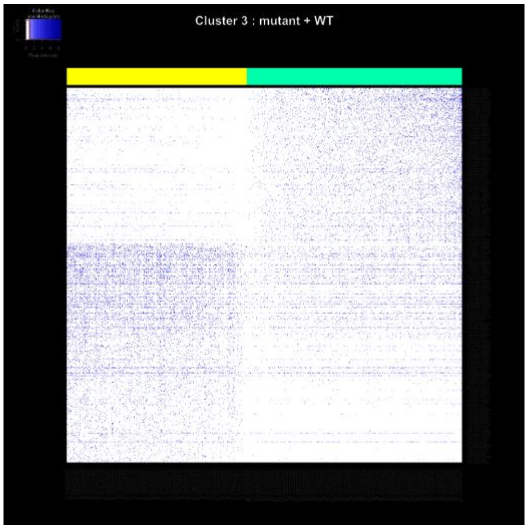
Cluster 3 : mutant + WT

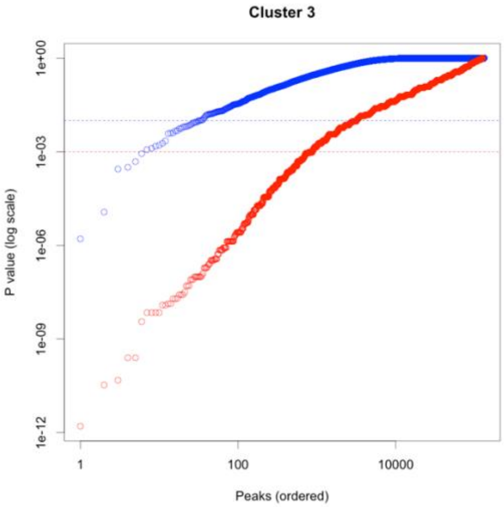
Cluster 3
1e+00
1e-03
1e-06
1e-09
1e-12
P value (log scale)
1
100
10000
Peaks (ordered)

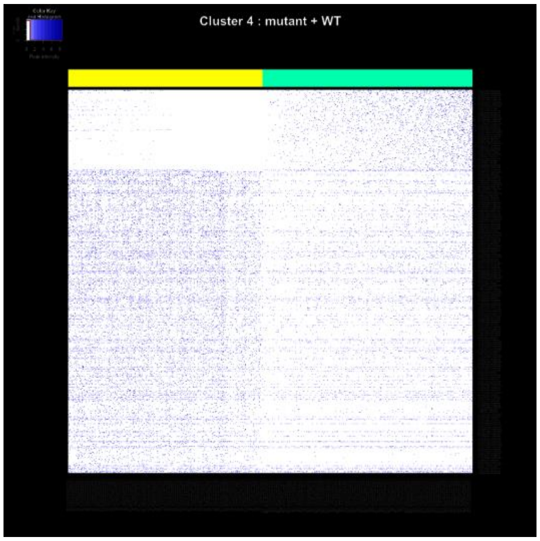
Cluster 4 : mutant + WT

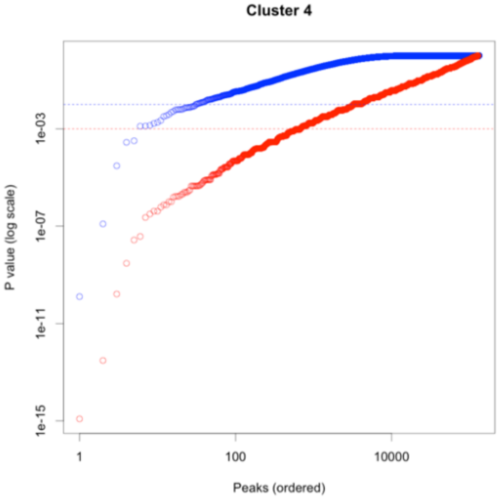
Cluster 4
1e-03
1e-07
1e-11
1e-15
P value (log scale)
1
100
10000
Peaks (ordered)

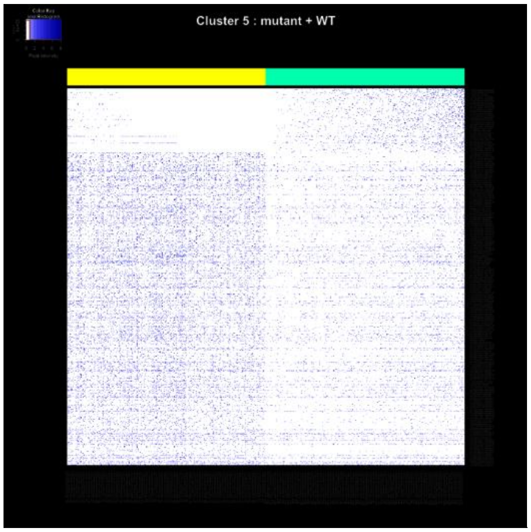
Cluster 5 : mutant + WT

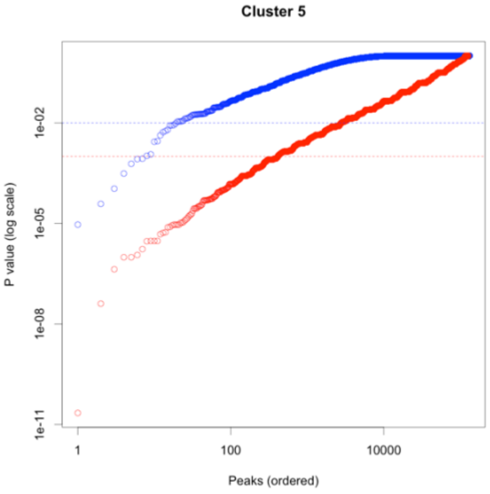
Cluster 5
P value (log scale)
1e-02
1e-05
1e-08
1e-11
1
100
10000
Peaks (ordered)

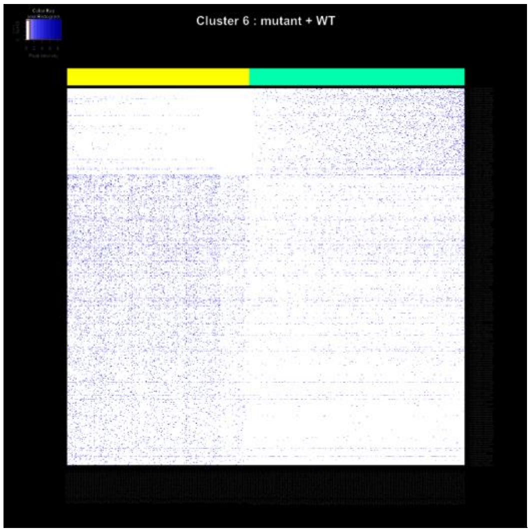
Cluster 6 : mutant + WT

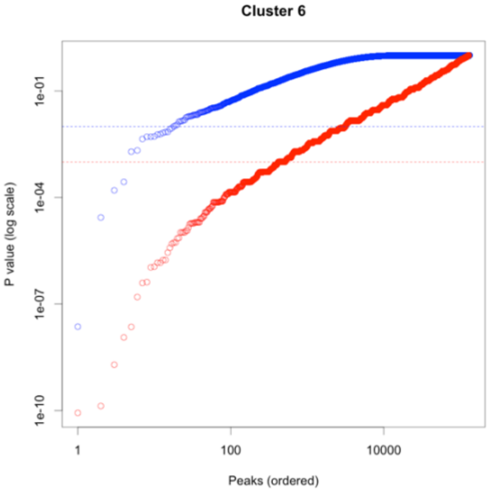
Cluster 6
P value (log scale)
1e-01
1e-04
1e-07
1e-10
1
100
10000
Peaks (ordered)

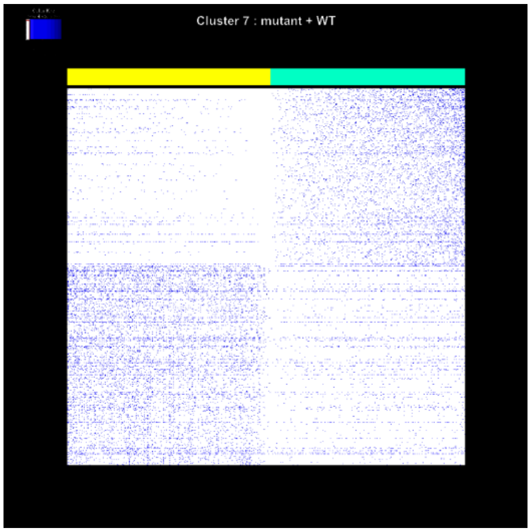
Cluster 7 : mutant + WT

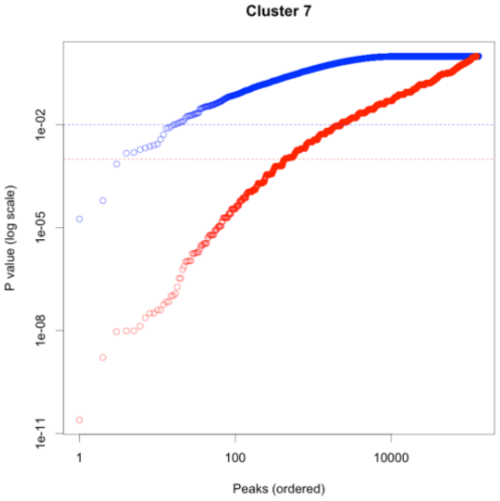
Cluster 7
P value (log scale)
1e-02
1e-05
1e-08
1e-11
1
100
10000
Peaks (ordered)

Cluster 8

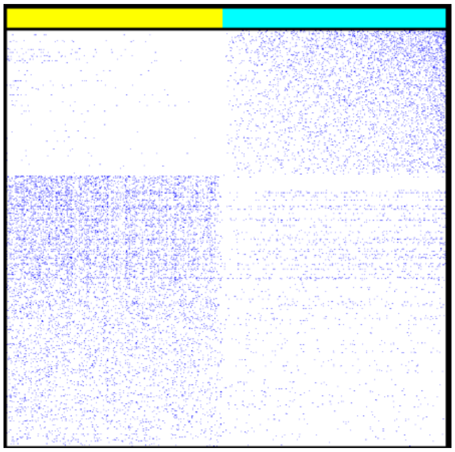

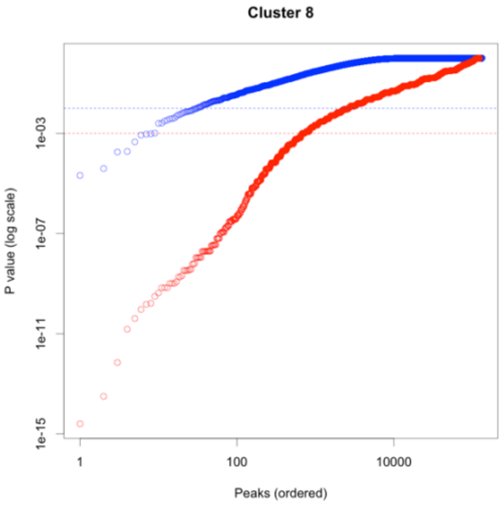
Cluster 8
P value (log scale)
1e-03
1e-07
1e-11
1e-15
1
100
10000
Peaks (ordered)

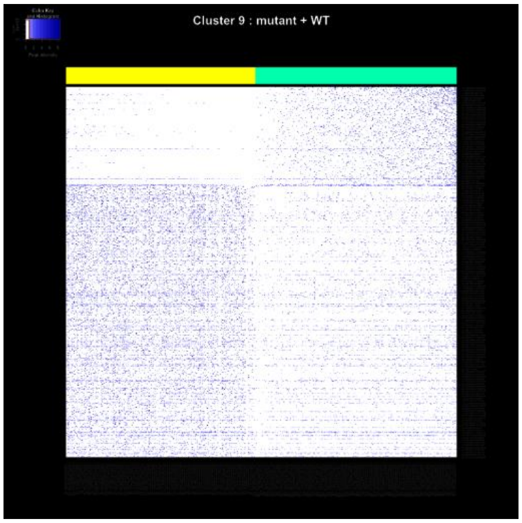
Cluster 9 : mutant + WT

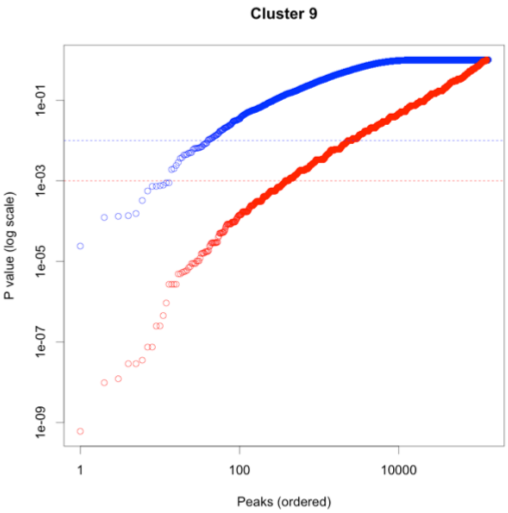
Cluster 9
P value (log scale)
1e-01
1e-03
1e-05
1e-07
1e-09
1
100
10000
Peaks (ordered)

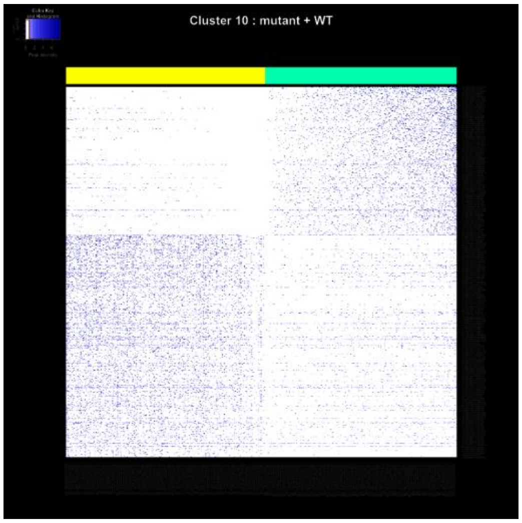
Cluster 10 : mutant + WT

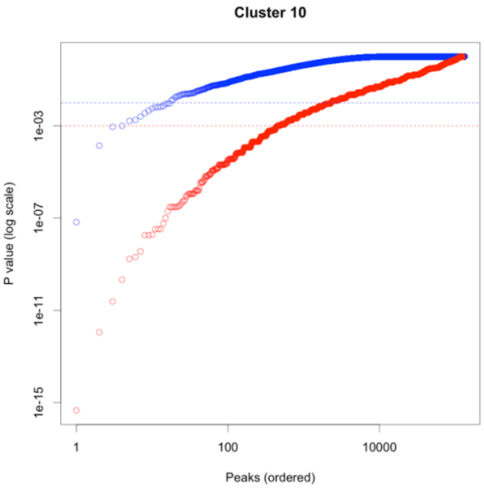
Cluster 10
P value (log scale)
1e-03
1e-07
1e-11
1e-15
1
100
10000
Peaks (ordered)

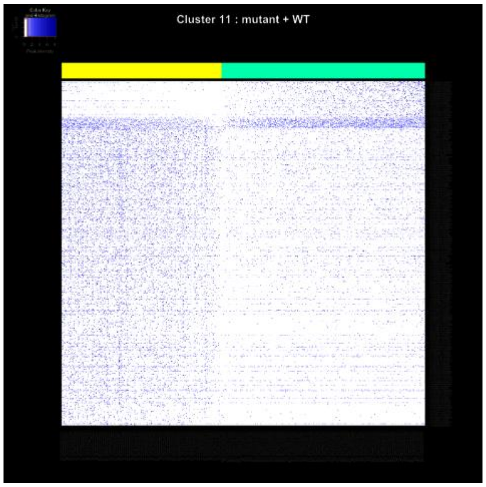
Cluster 11 : mutant + WT

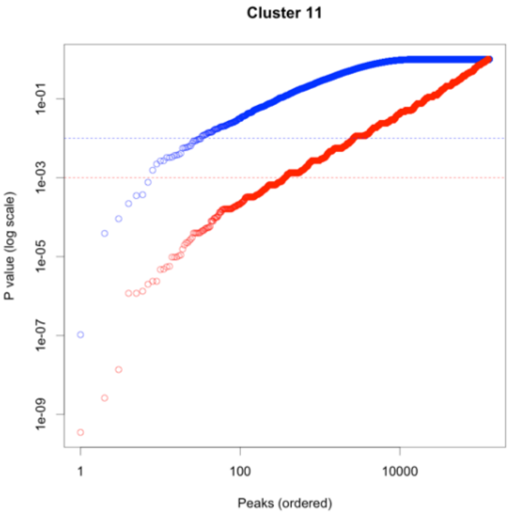
Cluster 11
P value (log scale)
1e-01
1e-03
1e-05
1e-07
1e-09
1
100
10000
Peaks (ordered)

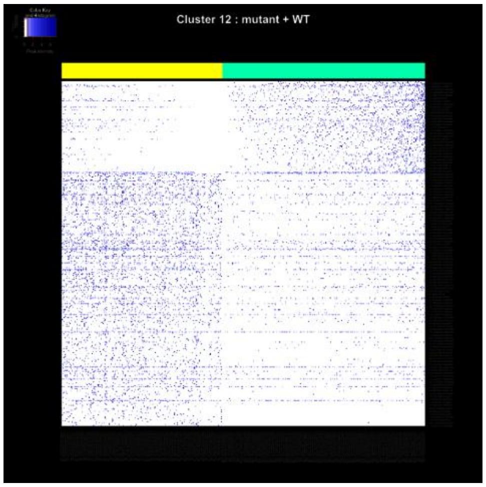
Cluster 12 : mutant + WT

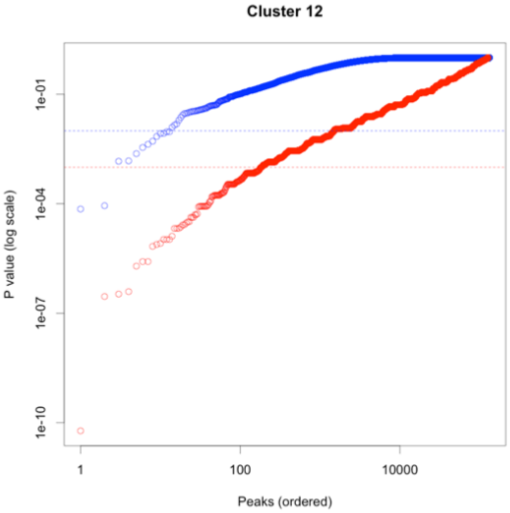
Cluster 12
P value (log scale)
1e-01
1e-04
1e-07
1e-10
1
100
10000
Peaks (ordered)

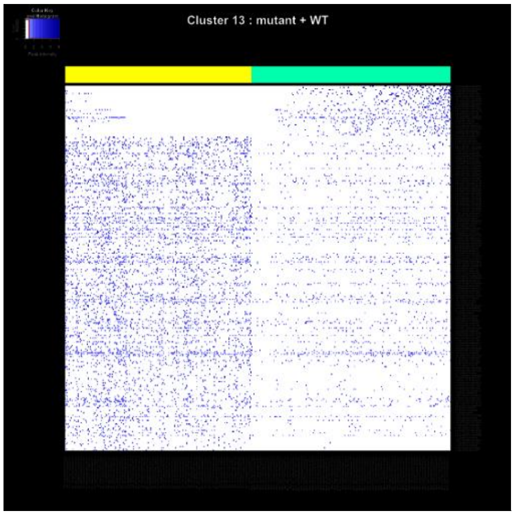
Cluster 13 : mutant + WT

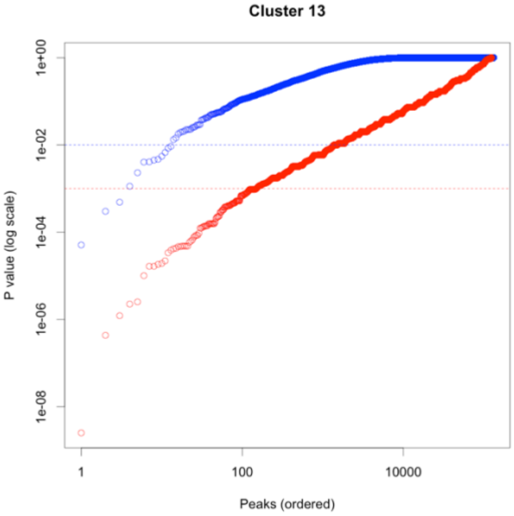
Cluster 13
P value (log scale)
1e+00
1e-02
1e-04
1e-06
1e-08
1
100
10000
Peaks (ordered)

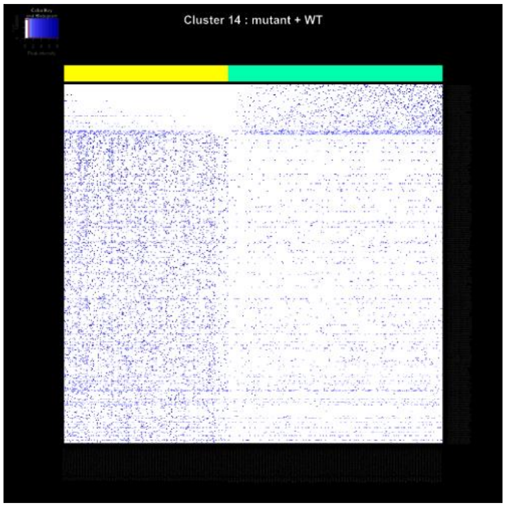
Cluster 14 : mutant + WT

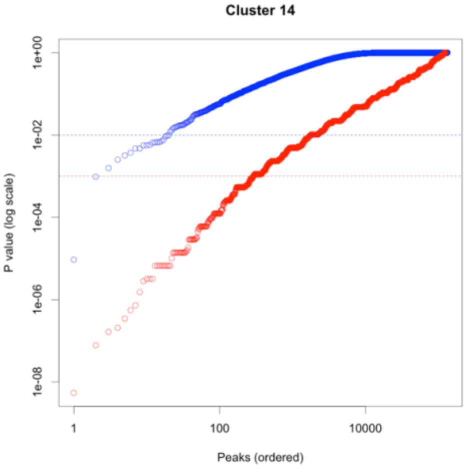
Cluster 14
P value (log scale)
1e+00
1e-02
1e-04
1e-06
1e-08
1
100
10000
Peaks (ordered)

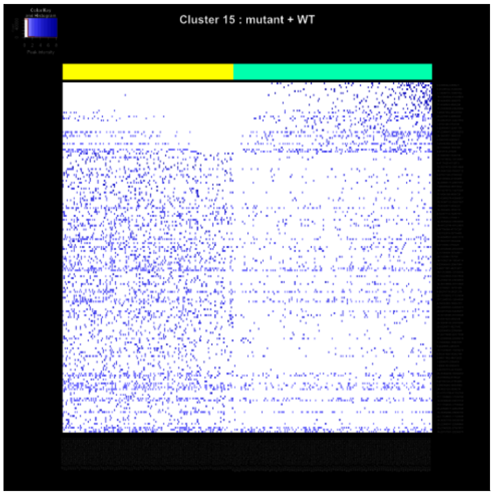
Cluster 15 : mutant + WT

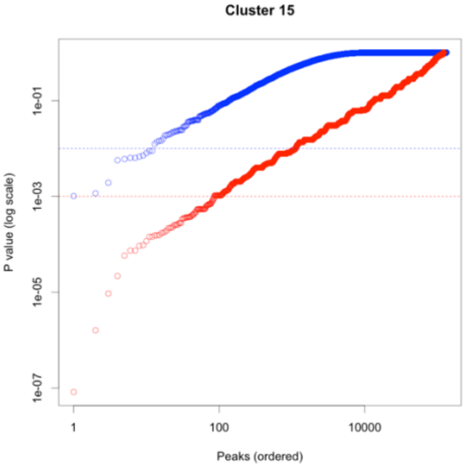
Cluster 15
P value (log scale)
1e-01
1e-03
1e-05
1e-07
1
100
10000
Peaks (ordered)

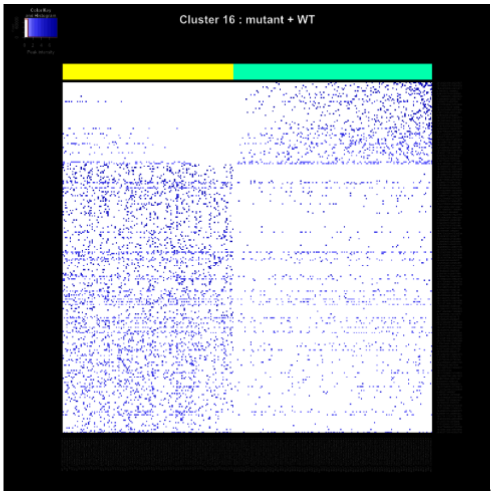
Cluster 16 : mutant + WT

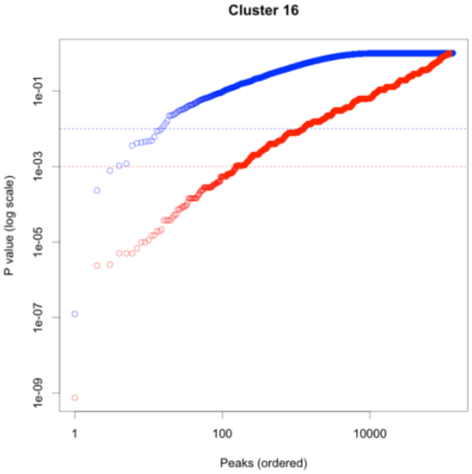
Cluster 16
P value (log scale)
1e-01
1e-03
1e-05
1e-07
1e-09
1
100
10000
Peaks (ordered)

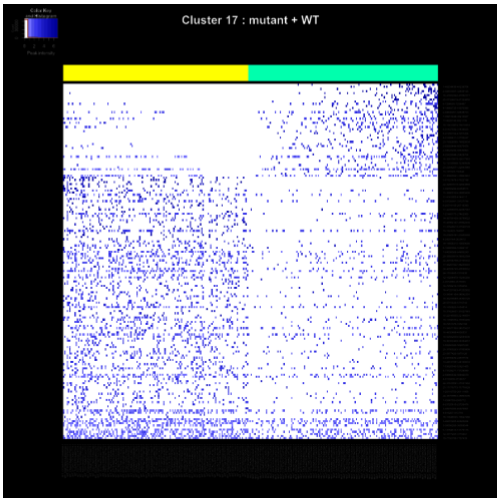
Cluster 17 : mutant + WT

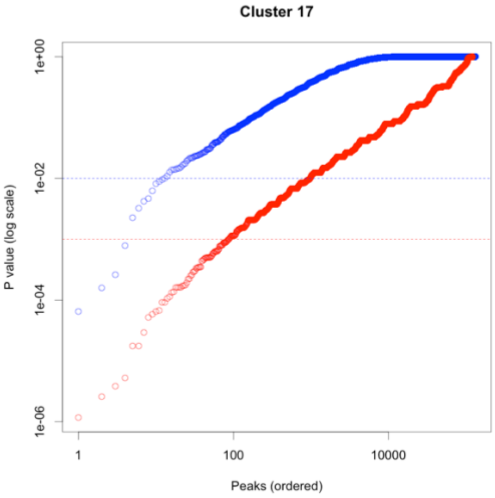
Cluster 17
P value (log scale)
1e+00
1e-02
1e-04
1e-06
1
100
10000
Peaks (ordered)

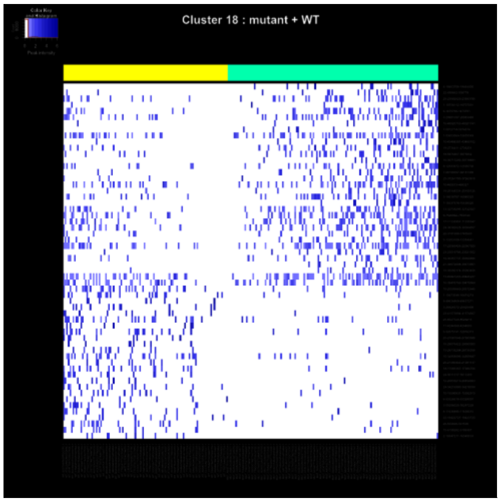
Cluster 18 : mutant + WT

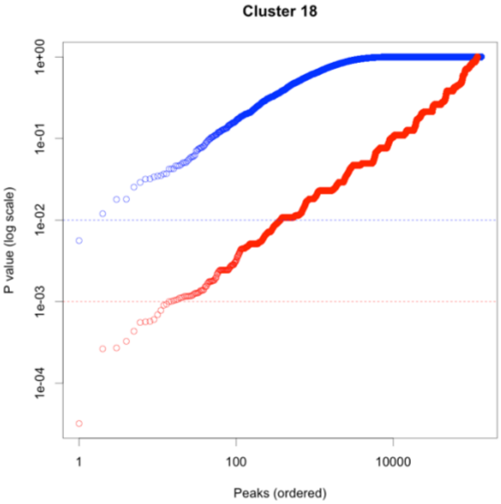
Cluster 18
P value (log scale)
1e+00
1e-01
1e-02
1e-03
1e-04
1
100
10000
Peaks (ordered)

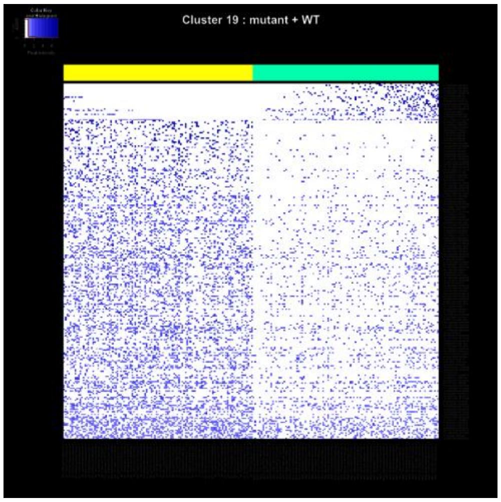
Cluster 19 : mutant + WT

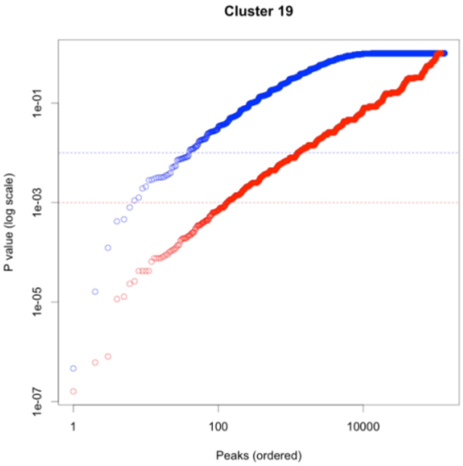
Cluster 19
P value (log scale)
1e-01
1e-03
1e-05
1e-07
1
100
10000
Peaks (ordered)

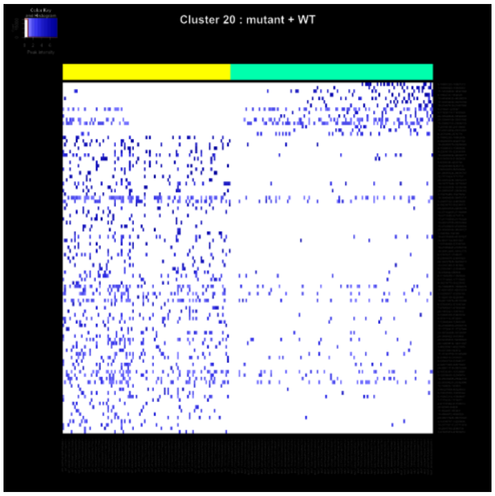
Cluster 20 : mutant + WT

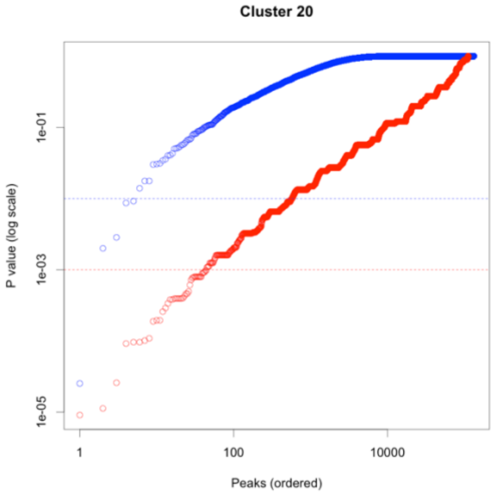
Cluster 20
P value (log scale)
1e-01
1e-03
1e-05
1
100
10000
Peaks (ordered)

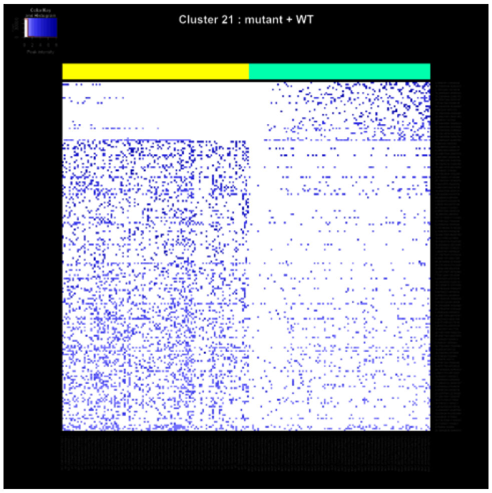
Cluster 21 : mutant + WT

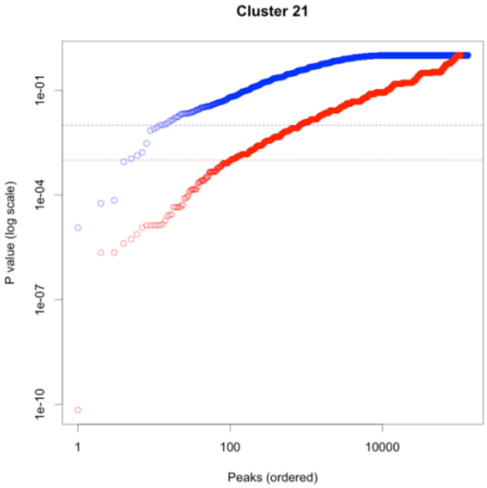
Cluster 21
P value (log scale)
1e-01
1e-04
1e-07
1e-10
1
100
10000
Peaks (ordered)

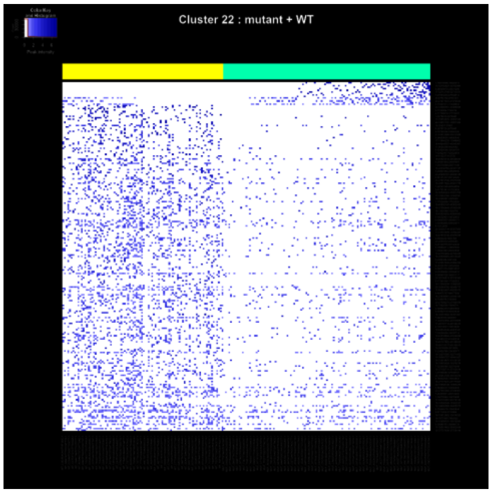
Cluster 22 : mutant + WT

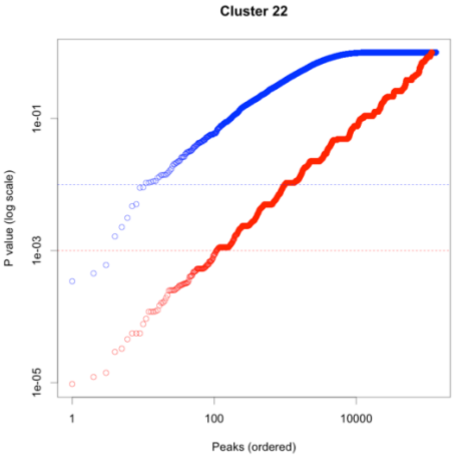
Cluster 22
P value (log scale)
1e-01
1e-03
1e-05
1
100
10000
Peaks (ordered)

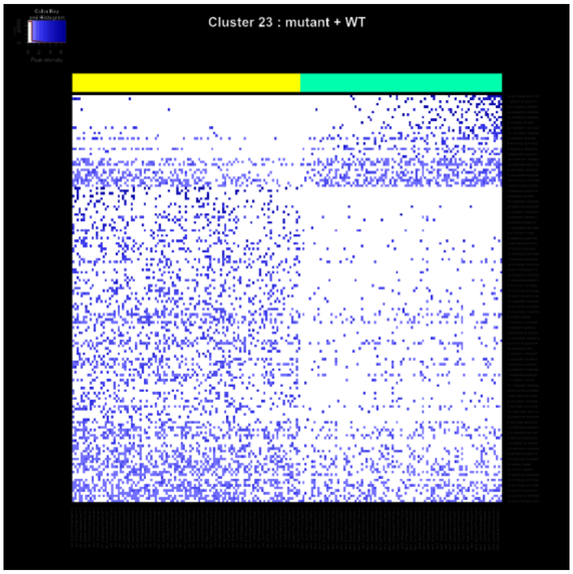
Cluster 23 : mutant + WT

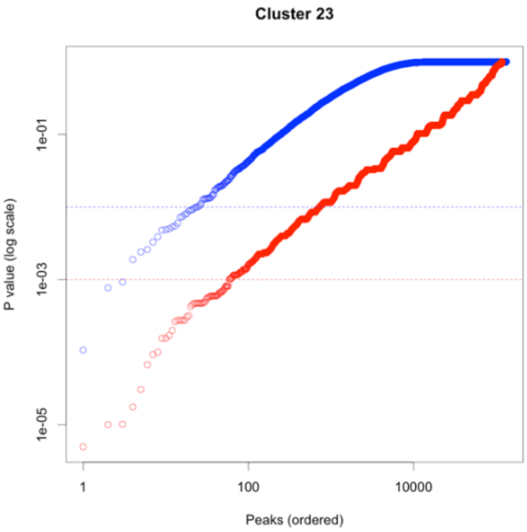
Cluster 23
P value (log scale)
1e-01
1e-03
1e-05
1
100
10000
Peaks (ordered)

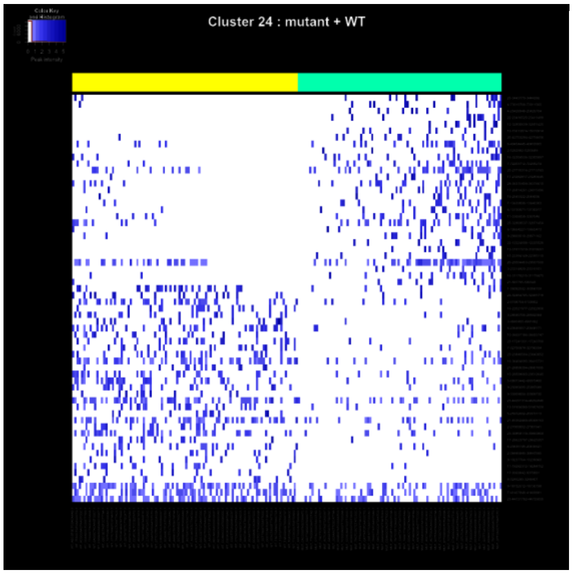
Cluster 24 : mutant + WT

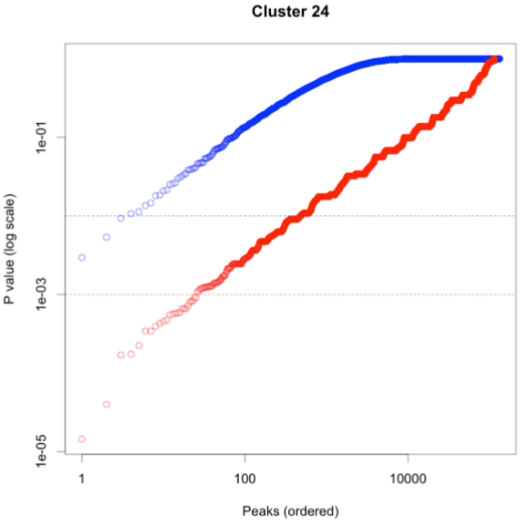
Cluster 24
P value (log scale)
1e-01
1e-03
1e-05
1
100
10000
Peaks (ordered)

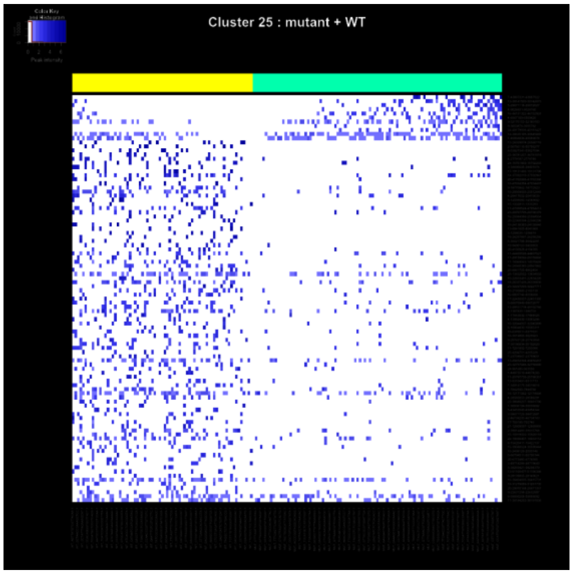
Cluster 25 : mutant + WT

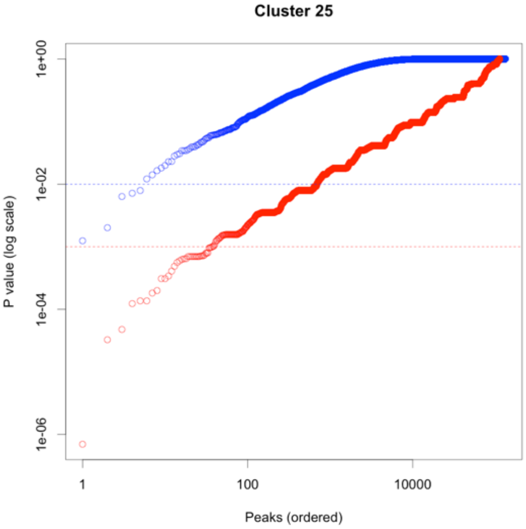
Cluster 25
P value (log scale)
1e+00
1e-02
1e-04
1e-06
1
100
10000
Peaks (ordered)

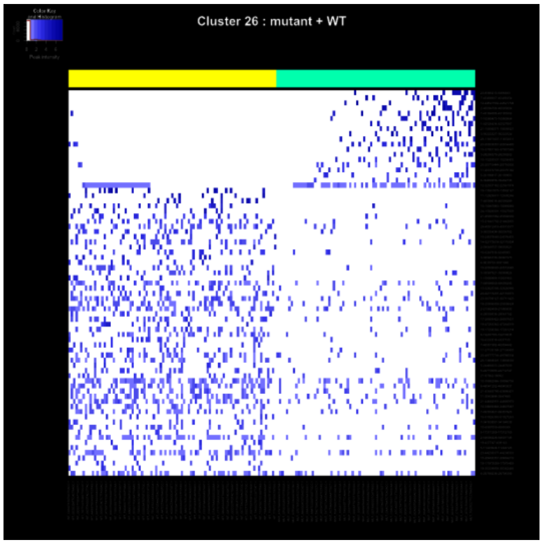
Cluster 26 : mutant + WT

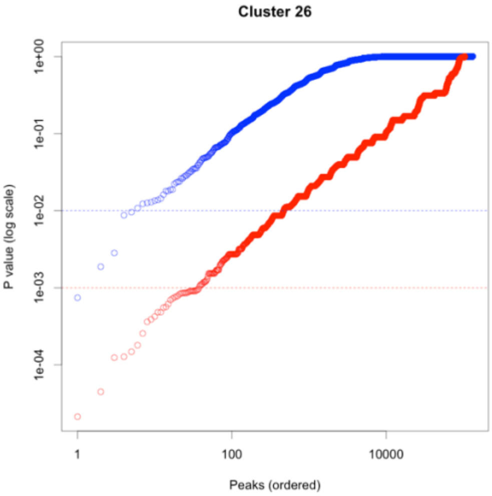
Cluster 26
P value (log scale)
1e+00
1e-01
1e-02
1e-03
1e-04
1
100
10000
Peaks (ordered)

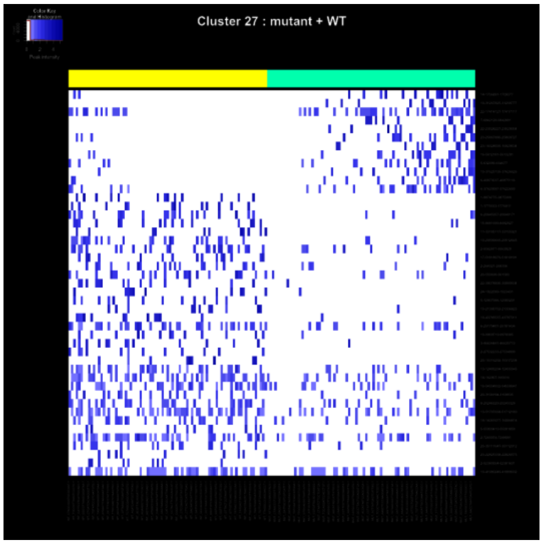
Cluster 27 : mutant + WT

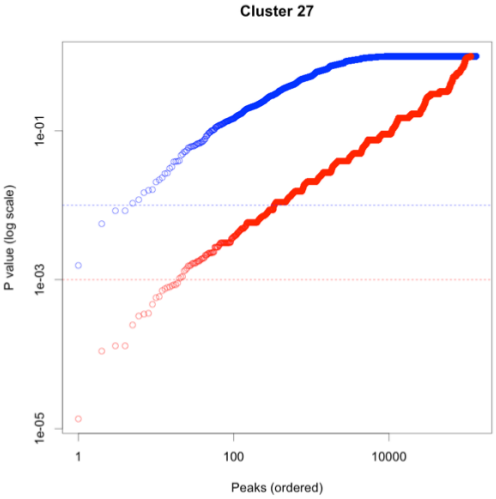
Cluster 27
P value (log scale)
1e-01
1e-03
1e-05
1
100
10000
Peaks (ordered)

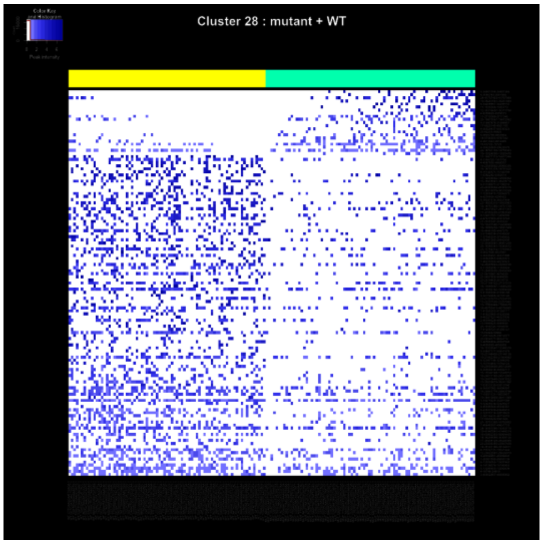
Cluster 28 : mutant + WT

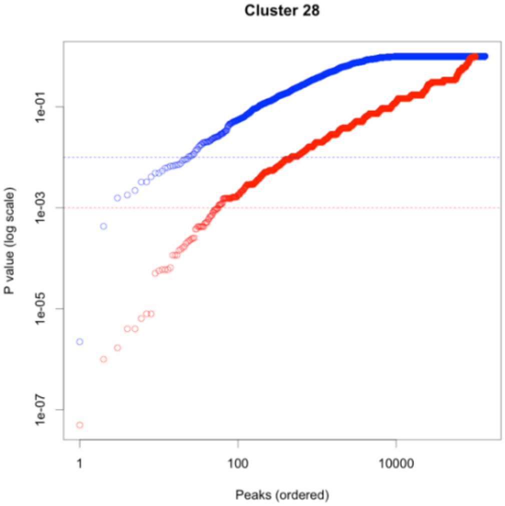
Cluster 28
P value (log scale)
1e-01
1e-03
1e-05
1e-07
1
100
10000
Peaks (ordered)

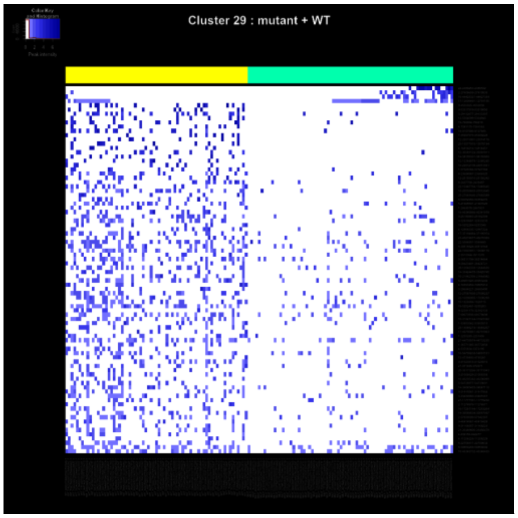
Cluster 29 : mutant + WT

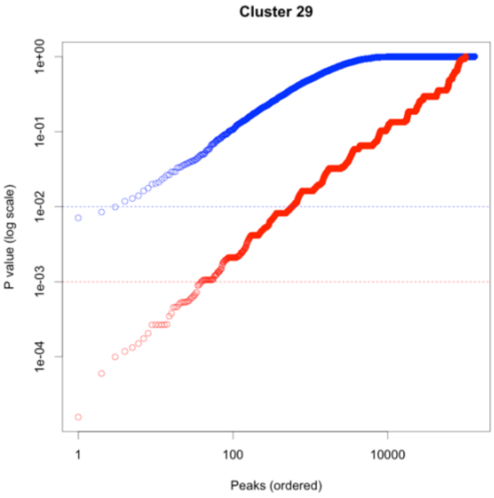
Cluster 29
1e+00
1e-01
1e-02
1e-03
1e-04
P value (log scale)
1
100
10000
Peaks (ordered)

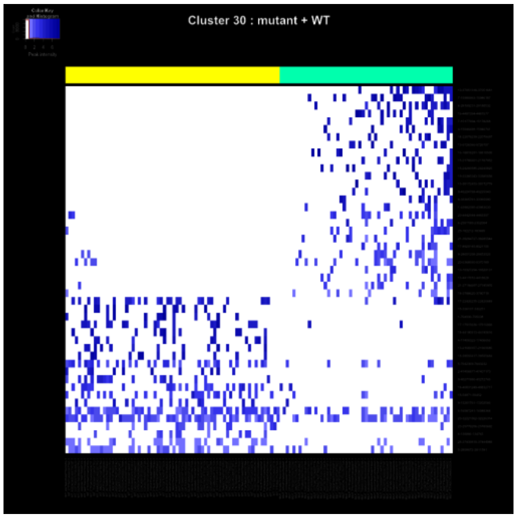
Cluster 30 : mutant + WT

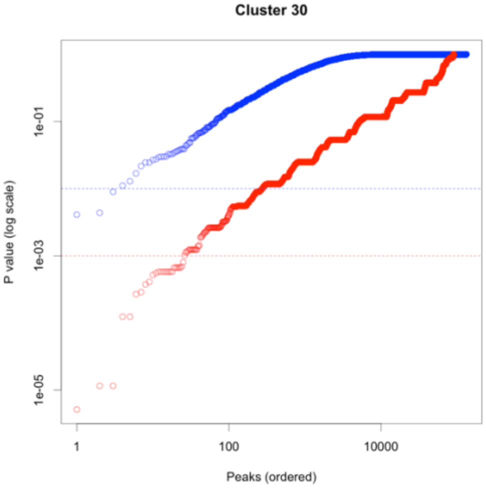
Cluster 30
1e-01
1e-03
1e-05
P value (log scale)
1
100
10000
Peaks (ordered)

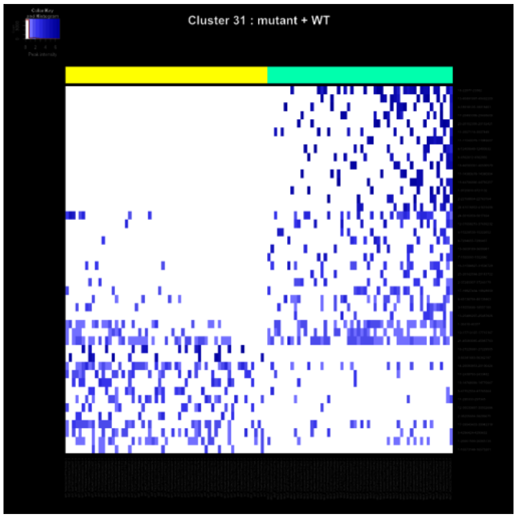
Cluster 31 : mutant + WT

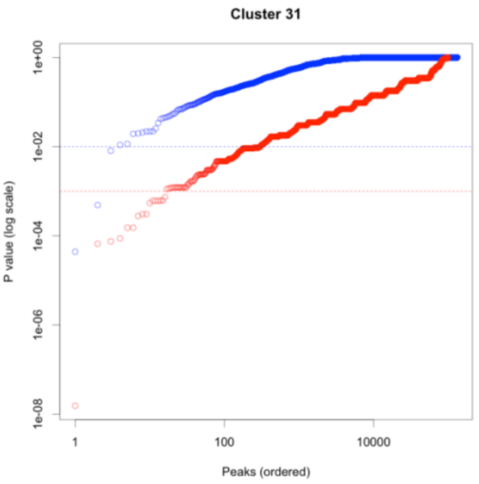
Cluster 31
1e+00
1e-02
1e-04
1e-06
1e-08
P value (log scale)
1
100
10000
Peaks (ordered)

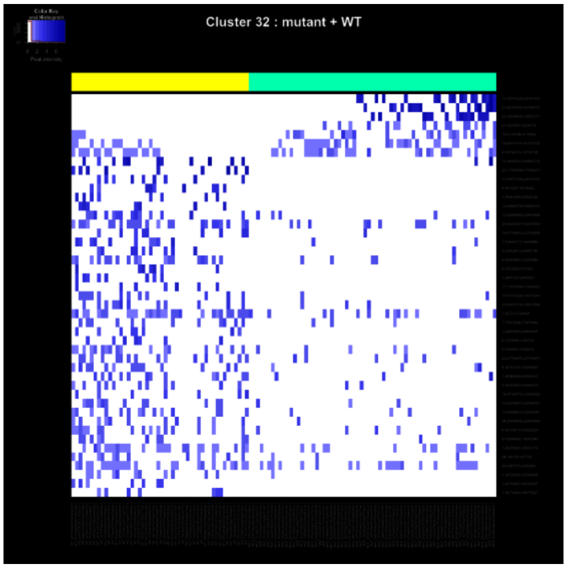
Cluster 32 : mutant + WT

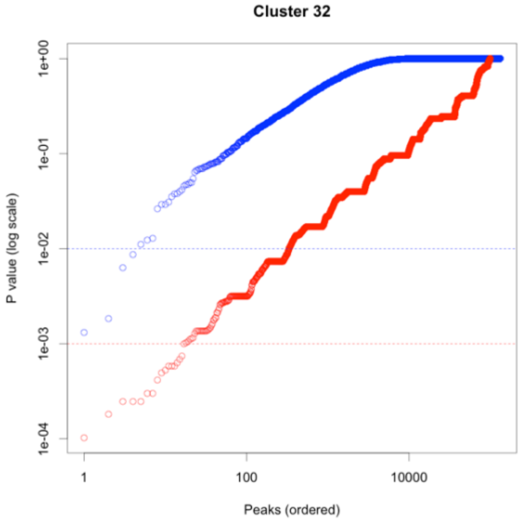
Cluster 32
P value (log scale)
1e+00
1e-01
1e-02
1e-03
1e-04
1
100
10000
Peaks (ordered)

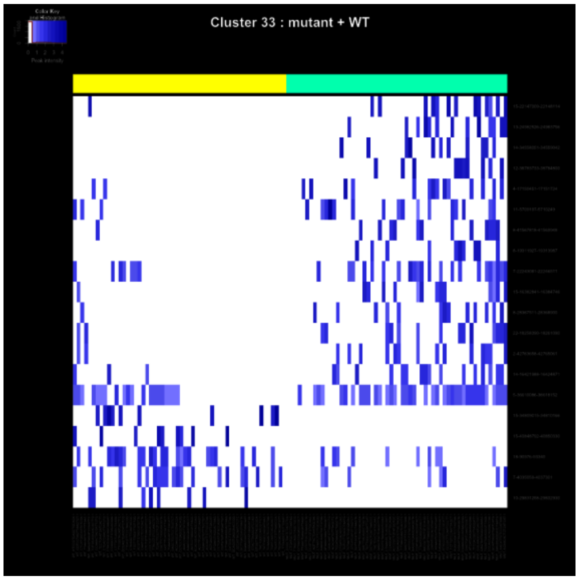
Cluster 33 : mutant + WT

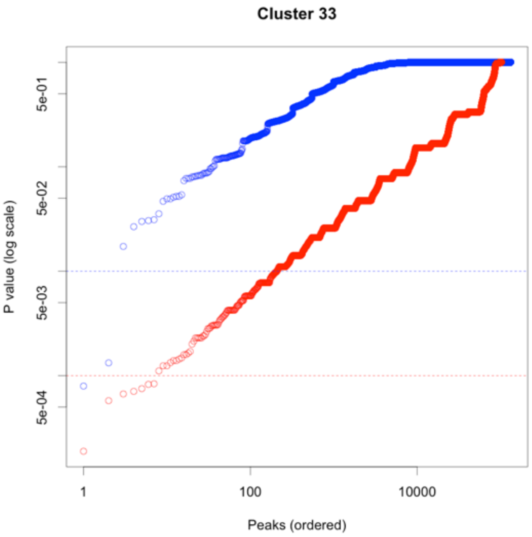
Cluster 33
P value (log scale)
5e-01
5e-02
5e-03
5e-04
1
100
10000
Peaks (ordered)

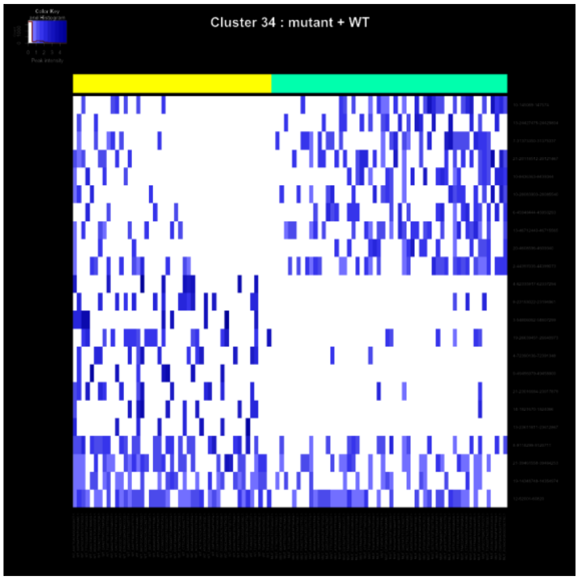
Cluster 34 : mutant + WT

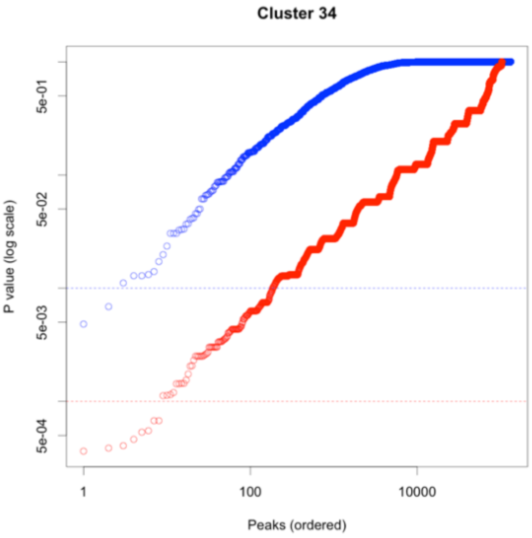
Cluster 34
P value (log scale)
5e-01
5e-02
5e-03
5e-04
1
100
10000
Peaks (ordered)

Cluster 35

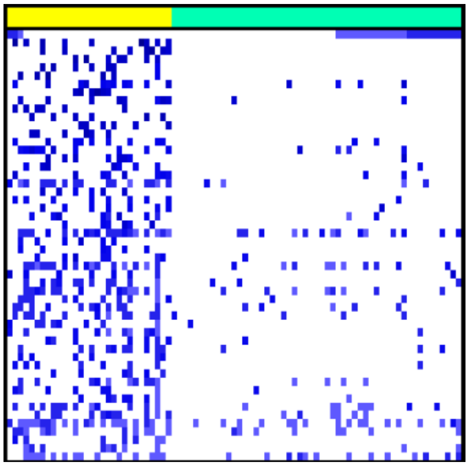

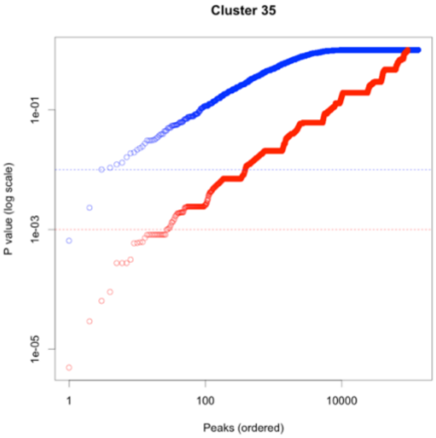
Cluster 35
P value (log scale)
1e-01
1e-03
1e-05
1
100
10000
Peaks (ordered)

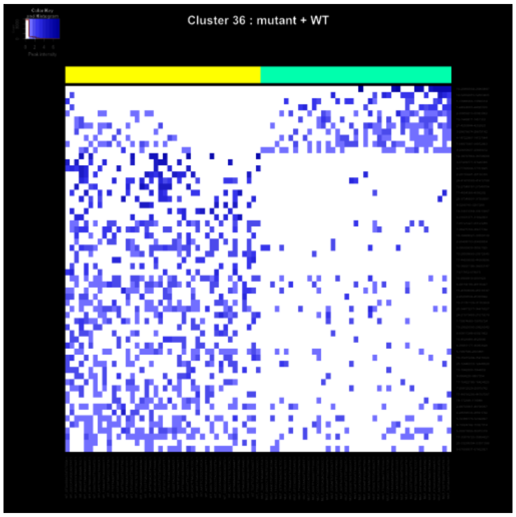
Cluster 36 : mutant + WT

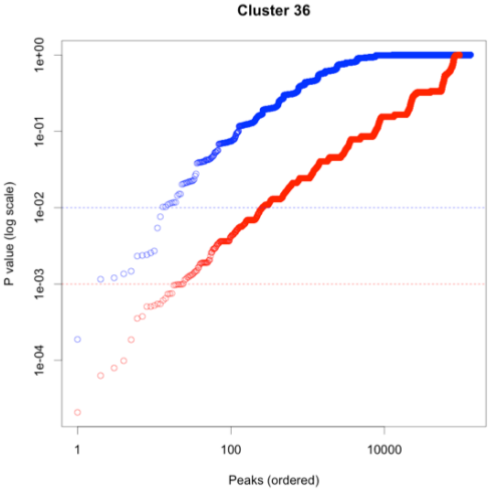
Cluster 36
P value (log scale)
1e+00
1e-01
1e-02
1e-03
1e-04
1
100
10000
Peaks (ordered)

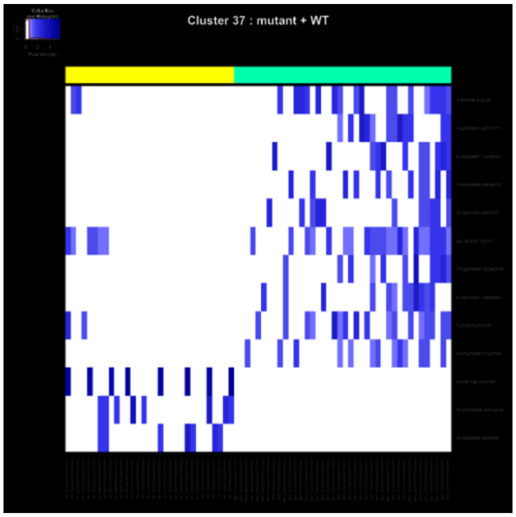
Cluster 37 : mutant + WT

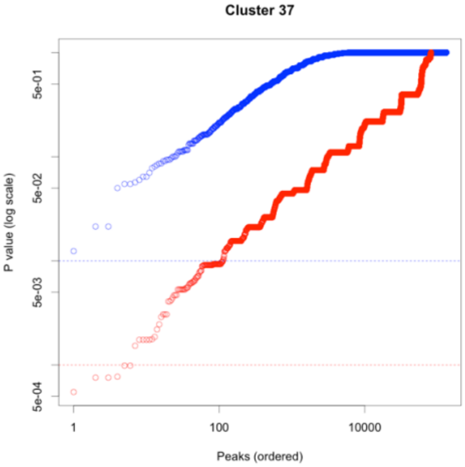
Cluster 37
P value (log scale)
5e-01
5e-02
5e-03
5e-04
1
100
10000
Peaks (ordered)

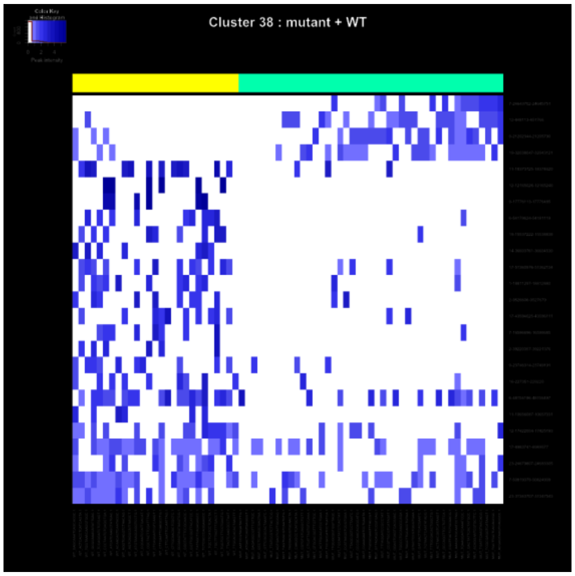
Cluster 38 : mutant + WT

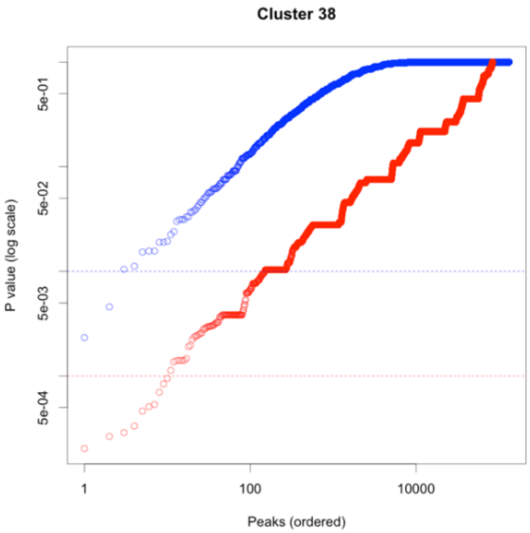
Cluster 38
P value (log scale)
5e-01
5e-02
5e-03
5e-04
1
100
10000
Peaks (ordered)

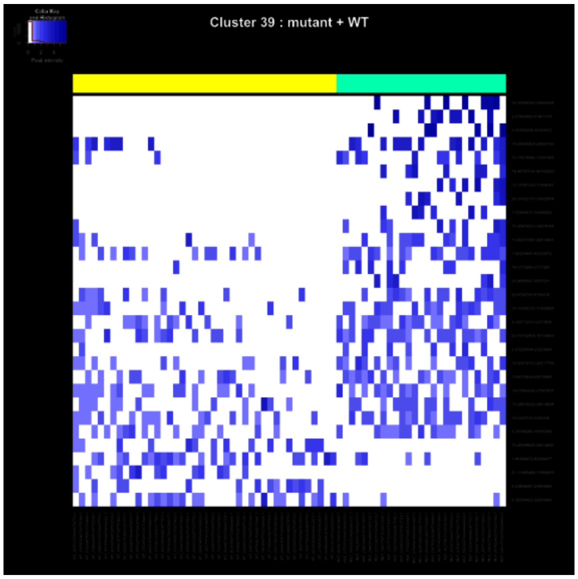
Cluster 39 : mutant + WT

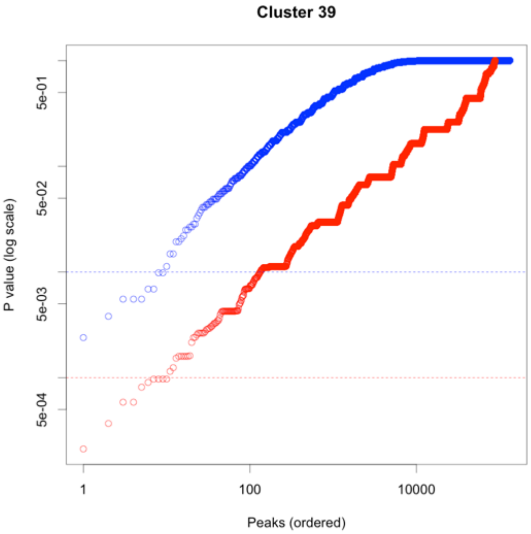
Cluster 39
P value (log scale)
5e-01
5e-02
5e-03
5e-04
1
100
10000
Peaks (ordered)

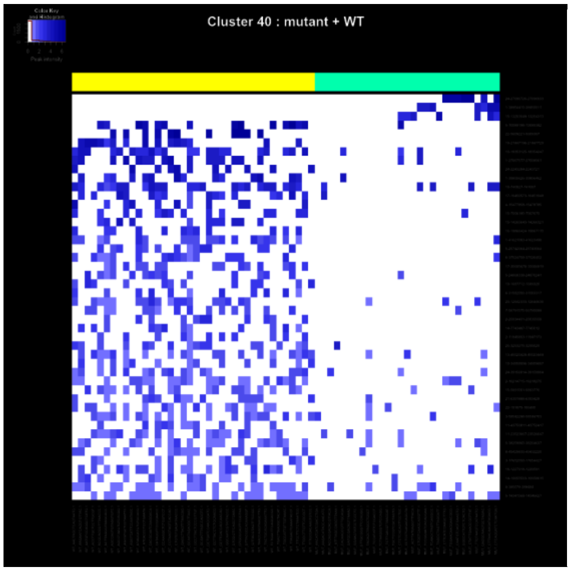
Cluster 40 : mutant + WT

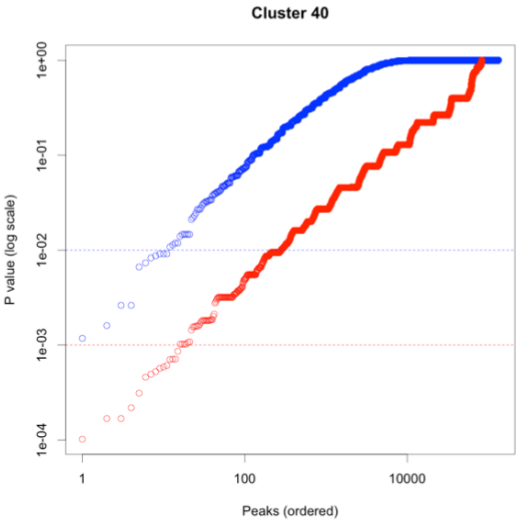
Cluster 40
P value (log scale)
1e+00
1e-01
1e-02
1e-03
1e-04
1
100
10000
Peaks (ordered)

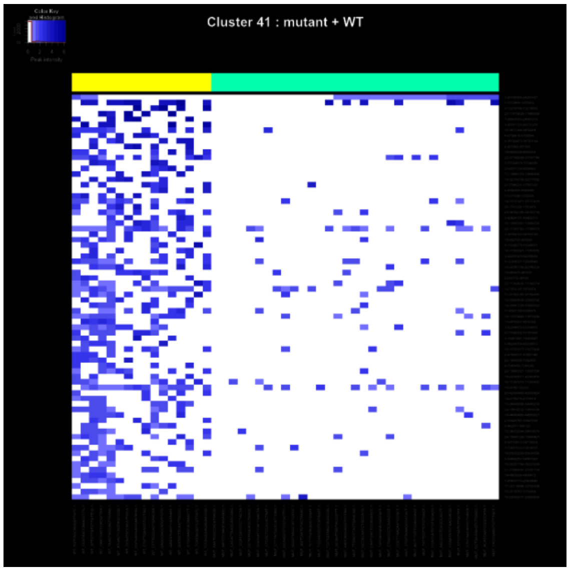
Cluster 41 : mutant + WT

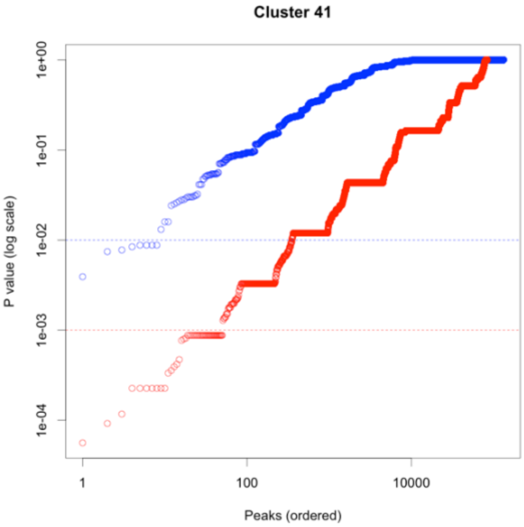
Cluster 41
P value (log scale)
1e+00
1e-01
1e-02
1e-03
1e-04
1
100
10000
Peaks (ordered)

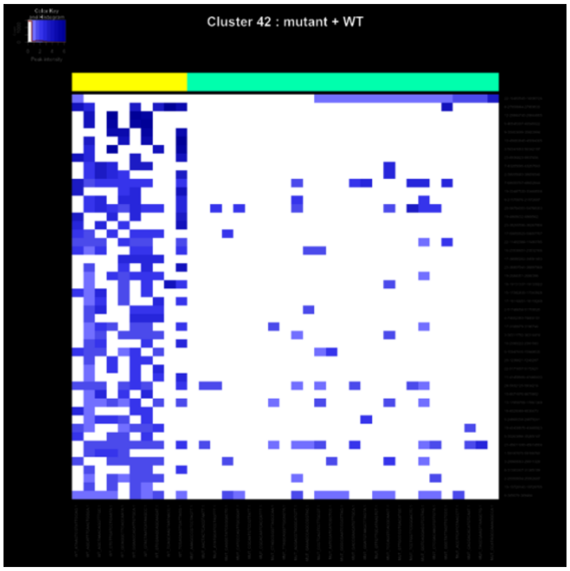
Cluster 42 : mutant + WT

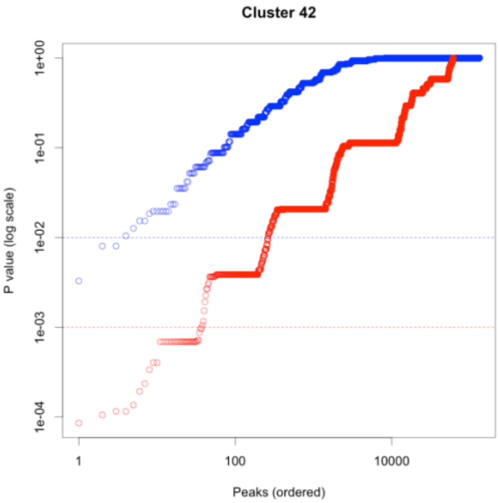
Cluster 42
P value (log scale)
1e+00
1e-01
1e-02
1e-03
1e-04
1
100
10000
Peaks (ordered)
